# Targeting Aggressive Prostate Carcinoma Cells with Mesothelin-CAR-T Cells

**DOI:** 10.3390/biomedicines13051215

**Published:** 2025-05-16

**Authors:** Apolline de Testas de Folmont, Angèle Fauvel, Francis Vacherot, Pascale Soyeux, Abdérémane Abdou, Salem Chouaib, Stéphane Terry

**Affiliations:** 1 INSERM UMR 1186, Integrative Tumor Immunology and Immunotherapy, Gustave Roussy, University Paris-Saclay, 94805 Villejuif, France; apolline.de-testas-de-folmont@curie.fr (A.d.T.d.F.); angelefauvel@gmail.com (A.F.); abderamane.abdou@gustaveroussy.fr (A.A.); salem.chouaib@gustaveroussy.fr (S.C.); 2 INSERM U 932, Institut Curie, PSL Research University, 75005 Paris, France; 3 TRePCa, Université Paris Est Créteil, 94010 Creteil, France; vacherot@u-pec.fr (F.V.); soyeux@u-pec.fr (P.S.); 4 Thumbay Research Institute for Precision Medicine, Gulf Medical University, Ajman 4184, United Arab Emirates; 5 Research Department, Inovarion, 75005 Paris, France

**Keywords:** prostate cancer, aggressive, metastatic cancer, epithelial–mesenchymal plasticity, CAR-T, mesothelin, adoptive cell immunotherapy, hypoxia, T cell, cell-mediated cytotoxicity

## Abstract

**Background**: Advancing chimeric antigen receptor (CAR) T cell therapy for solid tumors remains a major challenge in cancer immunotherapy. Prostate cancer (PCa), particularly in its aggressive forms, may be a suitable target for CAR-T therapy given the range of associated tumor antigens. However, due to the high plasticity and heterogeneity of aggressive PCa and the complexity of the tumor environment, there is a need to broaden the repertoire of targetable antigens and deepen our understanding of CAR-T behavior in stressed microenvironmental conditions. Growing evidence supports mesothelin as a promising cancer-associated marker and a compelling target for CAR-T cell approaches in solid tumors. **Objectives and Methods**: Here, we employed gene expression datasets to investigate mesothelin expression in both primary and metastatic PCa tumors. Additionally, we evaluated mesothelin expression across various preclinical PCa models and assessed the therapeutic efficacy of second-generation mesothelin-targeted CAR-T (meso-CAR-T) cells under both normoxic and hypoxic conditions, with hypoxia as a representative tumor-associated stress condition. **Results**: Our results revealed a significant enrichment of mesothelin in 3–10% of metastatic prostate tumors, contrasting with its minimal expression in primary tumors. In line with these findings, we observed increased mesothelin expression in an aggressive variant of the 22Rv1 cell line, which displayed an epithelial–mesenchymal plasticity (EMP) phenotype. Meso-CAR-T cells demonstrated potent cytotoxicity and remarkable selectivity toward these carcinoma cells under both severe hypoxia (1% O_2_) or normoxia (21% O_2_), highlighting their ability to withstand metabolic stress within the tumor microenvironment. **Conclusions**: Our study underscores the potential of meso-CAR-T cells as a promising strategy for targeting specific subtypes of metastatic prostate cancer.

## 1. Introduction

Prostate cancer (PCa) remains one of the leading causes of cancer-related deaths in men [1]. While localized and regional diseases are generally well controlled by current therapies, not all patients achieve a cure. Over the past decade, novel treatments that inhibit the androgen receptor and its downstream pathways have extended the lives of men with advanced prostate cancer. However, metastatic PCa ultimately develops resistance to these therapies [2,3,4]. Patients with metastatic disease have a five-year survival rate close to 30% [5,6]. Recent advancements have led to the development of various immunotherapeutic approaches for both liquid and solid tumors, including immune checkpoint inhibitors, vaccine-based therapies, and cell adoptive transfer approaches, such as the adoptive transfer of chimeric antigen receptor (CAR)-T cells targeting specific tumor antigens [7,8,9].

Patients with metastatic castration-resistant prostate cancer (CRPC) may benefit from Sipuleucel-T, which involves the injection of dendritic cells producing a fusion protein (PA2024) comprising prostatic acid phosphatase (PAP) and granulocyte-macrophage colony-stimulating factor (GM-CSF) to stimulate dendritic cell maturation [10], but the benefits are limited to a few months. The use of immune checkpoint inhibitors has shown some promising results in small subgroups of patients with a particular molecular profile [11]. However, overall, metastatic CPRC patients are refractory to this treatment [12,13], and no other immunotherapy options are currently available for these patients. Other strategies, such as bispecific T cell engagers (BiTEs) [14,15] and CAR-T cells, show promise but are not yet established as standard treatments [16,17,18,19].

CAR-T cells interact with tumor cells expressing specific tumor-associated antigens independently of the major histocompatibility complex (MHC) peptides, leading to the destruction of the target tumor cell [20,21]. Despite their increasing use in treating B-cell malignancies, several challenges limit the application of CAR-T cells in solid tumors: (1) tumor antigen heterogeneity, (2) tumor antigen specificity and potential toxicities, (3) trafficking of CAR-T cells to the tumor site, (4) overcoming the immunosuppressive microenvironment, and (5) persistence of CAR-T cells over time [20,21].

In this context, hypoxia, a low-oxygen condition that can accompany tumor development, may also play a role. Tumor hypoxia has been proposed to contribute to the immunosuppressive microenvironment and immune escape, as well as tumor heterogeneity [22,23,24,25,26]. Hypoxia has been documented in prostate tumors, associating with poor prognosis and disease recurrence [27,28,29,30]. Hypoxia is often considered detrimental to CD8 T cell-mediated antitumor immunity [31], although this remains an open question as some studies have suggested beneficial functions of hypoxia and hypoxia-inducible factors for murine and human T cells [32,33,34]. Further research is needed to understand the impact of hypoxia on CAR-T cells.

An inherent challenge in developing therapeutic strategies, including CAR-T cell therapies, lies in their efficacy against aggressive, recurrent tumors that often lose differentiation, and associated tumor antigens. In PCa, considerable efforts have been dedicated to engineering CAR-T cells that target prostate-specific differentiation or cancer-related antigens [16,17,19,35], such as Prostate-Specific Membrane Antigen (PSMA) [36,37,38,39,40], Epithelial Cell Adhesion Molecule (EPCAM) [41], Prostate Stem Cell Antigen (PSCA) [42,43,44], and Six-Transmembrane Epithelial Antigen of Prostate 1 (STEAP1) [45,46,47]. However, in poorly differentiated and heavily treated tumors, these markers are often heterogeneous, expressed at low levels, or even absent, thereby presenting risks of intrinsic resistance or rapid disease recurrence. Thus, identifying additional target antigens is essential to expand the therapeutic range of CAR-T therapy in PCa.

Mesothelin has emerged as a compelling marker and target across multiple malignancies due to its high tumor specificity and elevated expression in advanced cancers [48,49,50,51]. Several clinical trials are already evaluating mesothelin-targeted CAR-T cells (known as meso-CAR-T, CART-meso or anti-MSLN CAR-T cells) in various cancer types [52]. However, the therapeutic potential of meso-CAR-T cells in PCa remains underexplored, partly due to limited knowledge on mesothelin expression in this cancer type, highlighting the need for further investigation [53,54].

In this study, we aimed to better characterize mesothelin expression in PCa and PCa models, and to evaluate the therapeutic potential of second-generation meso-CAR-T cells under both normoxic and hypoxic conditions, with hypoxia simulating a tumor-associated stress. We found that mesothelin was upregulated in a subset of metastatic PCa tumors but largely absent in primary PCa tumors. Furthermore, high levels of mesothelin were detected in an aggressive variant of 22Rv1 PCa cells, which lacks androgen receptor signaling and other typical PCa markers, while exhibiting an epithelial–mesenchymal plasticity (EMP) phenotype. Meso-CAR-T cells demonstrated robust tumor-specific cytotoxicity and high target specificity under both hypoxic and normoxic conditions, thus reflecting their metabolic resilience to tumor environmental stress. This data highlights the potential of meso-CAR-T cells as a promising therapeutic strategy for targeting specific metastatic PCa subtypes with reduced epithelial characteristics.

## 2. Materials and Methods

### 2.1. Cell Culture

Anti-Mesothelin (M11) h(41BB-CD3ζ) CAR-T cells and control non-transduced T cells were a kind gift from Dr. Carl H. June. The characteristics of the CAR and generation of the CAR-T cells was previously described [55,56]. Briefly, this CAR comprises an extracellular domain with an ScFv (single-chain fragment variable) of anti-mesothelin, a CD8a transmembrane domain, an intracellular signaling domain comprising a stimulatory domain, 4-1BB (also known as CD137), known to promote the persistence of CAR-T cells to significantly increases antitumor activity, and CD3ζ (also known as T cell receptor zeta), which plays an important role in coupling antigen recognition to several intracellular signal-transduction pathways. CAR M11 T cells were generated from T cells isolated from healthy volunteer donors. Purified T cells were activated by CD3 and CD28 beads, transduced and expanded as previously in a medium containing RPMI-1640, 10% Premium Fetal bovine serum (FBS), 1× Pen-strep, and 10 mM Hepes (Thermo Fisher Scientific, Waltham, MA, USA). The 22Rv1, DU145, PC3, VCaP, and LNCaP cell lines were obtained from the American Type Culture Collection (Manassas, VA, USA) and authenticated at this site. Cells were maintained in RPMI-1640 medium supplemented with 10% heat-inactivated FBS, 1% penicillin–streptomycin (Thermo Fisher Scientific). Unless indicated, cells were maintained at 37 °C in a 5% CO_2_ and 95% air (21% O_2_) incubator. The 22Rv1-CRIPTO cells were generated after stable transfection of CRIPTO (also known as TDGF1) using the CR-1 cDNA cloned into the p3XFLAG-Myc-CMV-25 expression vector (a kind gift from David S. Salomon) [57]. NCI-H660 (a kind gift from Mark Rubin) was cultivated in DMEM, and supplemented with 0.005 mg/mL Insulin, 10 nM Hydrocortisone (final conc.), 10 nM beta-estradiol (final conc.), 4 mM L-glutamine (for final conc. of 4 mM), 5% FBS. When indicated, hypoxia (1% O_2_) conditions were achieved using an InVivo2 400 Hypoxia Workstation (Ruskinn Technology, Bridgend, UK). In the different in vitro assays, the media were not supplemented with IL-2.

### 2.2. Analysis of Gene Expression Datasets Derived from PCa Patients

Gene expression data and associated sample information from various cohorts of human prostate cancer (PCa) were accessed on 23 May 2020, via cBioPortal https://www.cbioportal.org/ [58]. Cases with available expression data, disease state, and sample type information were considered, excluding cell lines and patient-derived xenograft (PDX) models. To compare *MSLN* expression across samples from independent cohorts, samples were classified based on automatically calculated z-scores. Z-score transformation normalized expression values by centering and scaling relative to the dataset mean and standard deviation, thereby standardizing gene expression across samples. *MSLN* expression was considered overexpressed in a given sample if its z-score exceeded +2, corresponding to approximately the 97.5th percentile under a standard normal distribution. This threshold balances sensitivity and specificity and aligns with previous studies demonstrating its suitability and statistical significance (*p* < 0.05) for microarray and transcriptomic data [59,60,61].

### 2.3. Cytotoxicity Assay

The cytotoxic activity of non-transduced and CAR-T cell clones was measured by a conventional 4 h ^51^Cr release assay as described [62,63], using Chromium-51 purchased from Perkin-Elmer (Waltham, MA, USA). Briefly, this was undertaken by co-culturing immune cells (Effector; E) and carcinoma cells (Target; T) at various E:T ratios for 4 h in round-bottomed 96-well plates using CAR-T medium. Specific lysis was calculated using the formula [(experimental CPM − spontaneous CPM)/total CPM] × 100. Results represent means of triplicates. For some cytotoxic assays, effector cells were preincubated for 40 h under normoxic or hypoxic conditions in the absence of antigen-positive carcinoma cells. Experimental workflow is shown in Supplementary Figure S1. For rechallenge assay, CAR-T cells were preincubated under normoxic or hypoxic conditions for 96 h in the presence of antigen-positive carcinoma cells at an E:T ratio of 3:1. CAR-T cells were then recovered and assayed in a standard ^51^Cr release assay. Experimental workflow is shown in Supplementary Figure S2. Antigen-naive CAR-T cells are used and tested for comparison purpose.

### 2.4. Tumor-Killing Assays

CAR-T cells and tumor targets were co-cultured at E:T ratio 1:5 in 6 well-plates for 0–48 h in carcinoma cell medium in the absence of exogenous IL-2 to assess antigen-driven responses, and analyzed by flow cytometry for CD45 expression on the cell surface. Tumor cell killing by CAR-T cells was assessed by measuring the percentage of CD45-negative cells at various time points and normalized to initial seeding levels. Experimental workflow is shown in Supplementary Figure S3.

### 2.5. Protein Preparation and Western Blot Analysis

Protein lysates were prepared as previously described [64]. Western blot analysis of MSLN was performed with mouse anti-MSLN (clone MN-1, 1/1000) from Sigma-Aldrich (St. Louis, MO, USA).

### 2.6. Immunohistochemistry and Immunofluorescence Stainings

Standard procedures were performed as previously described [65], but using as primary antibody anti-MSLN clone 5B2 at 1/5 dilution (Leica biosystems, Nussloch, Germany) for IHC, and clone MN1 at 1/500 for IF immunostaining.

### 2.7. RNA Preparation, cDNA Synthesis, and Quantitative Real-Time PCR

Total RNA extraction was performed using Trizol reagent. Reverse transcription was performed using Maxima Reverse Transcriptase followed by qPCR using real-time PCR Master SYBR Green on a StepOnePlus Real Time PCR system. Internal control genes included *RPLP0* and *HMBS*. All products were from Thermo Fisher Scientific. Primer sequences for the quantification were designed using Beacon Designer Free Edition and Primer3Plus (version: 3.3.0), purchased from Sigma-Aldrich (St. Louis, MO, USA), and are available upon request.

### 2.8. Flow Cytometry and Antibodies

Phenotypic analyses of carcinoma cells and immune cells were performed by direct immuno-staining. Briefly, 0.2 × 10^6^ cells were collected and resuspended in a FACS buffer (PBS with 2% FBS) and stained for 30 min in the dark; this process was performed at 4 °C with Abs for extracellular staining. Anti-CD3-BV711/Alexa700 (UCHT1), anti-CD69-APC-Cy7 (FN50), and anti-CCR7-FITC (3D12) were from BD Biosciences (Franklin Lakes, NJ, USA); anti-PD-1-PECy7 (eBioJ105) and anti-Ki67-PerCPefluor710 (20Raj1) were from Thermo Fisher Scientific. Anti-granzyme-B-APC/FITC (GB11), anti-CD4-AF700/APCcy7 (RPA-T4), anti-CD8-PacificBlue/PerCP Cy5.5 (RPA-T8), CD103-PE/Dazzle^TM^ 594 (Ber-ACT8), anti-TIM3-APC (F38-2E2), N-cadherin-APC (8C11), LAG3-FITC (7H2C65), and EPCAM-FITC (9C4) were from Biolegend (San Diego, CA, USA) Anti-CD45RA-APC/PE and anti-CD44-FITC (DB105) were purchased from Miltenyi Biotec (Bergisch Gladbach, Germany). Anti-mesothelin-APC (#420411) and anti-AXL-APC (#108724) were from R&D systems (Minneapolis, MN, USA). For intracellular staining (i.e., Ki67), cells were fixed/permeabilized with the ebioscience FoxP3 staining buffer set (Thermo Fisher Scientific) according to the manufacturer’s instructions, before staining directly conjugated antibodies in 1× Permeabilization Buffer for 30 min in the dark.

To detect dead cells, LIVE/DEAD^TM^ Fixable Far Red or Blue Dead Cell Stain Kits were used (Thermo Fisher Scientific). Acquisitions were performed using an LSR Fortessa (BD Biosciences) or a CYTOFLEX (Beckman Coulter, Brea, CA, USA) and data were processed using the FlowJo software v10.8.2 (BD Biosciences).

### 2.9. Statistical Analysis

Data analyses were performed using GraphPad Prism v8 (GraphPad Software, La Jolla, CA, USA) and Microsoft Excel (Microsoft Corp., Redmond, WA, USA). Group mean comparisons were conducted using the Mann–Whitney test or two-way ANOVA with multiple comparisons. Fisher’s exact test was used where applicable. All statistical tests were two-tailed, with α = 0.05 (* *p* ≤ 0.05).

## 3. Results

### 3.1. Mesothelin Expression Was Marginal in Primary Prostate Cancers but Upregulated in a Subset of Metastatic Prostate Cancers

**Figure 1:**
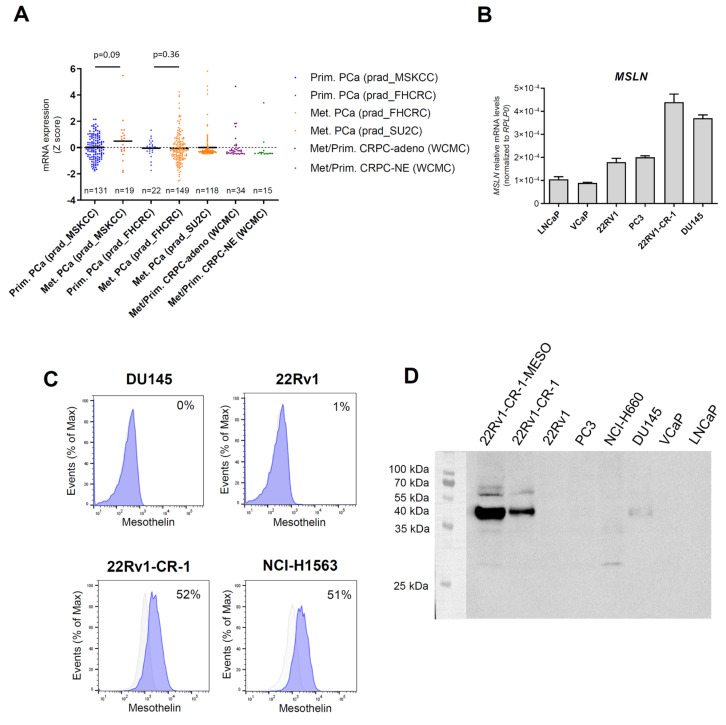
Mesothelin is expressed in metastatic PCa and in a PCa model with aggressive features. (**A**) *MSLN* mRNA levels in prostate tumor specimens from four patient cohorts containing primary and/or metastatic tumor samples. (**A**) Fisher’s exact test was applied to compare the proportion of *MSLN*-high cases between primary and metastatic samples. (**B**) *MSLN* mRNA levels in PCa cell lines, assessed by qRT-PCR. (**C**) Flow cytometric analysis of mesothelin expression in DU145, 22Rv1, 22Rv1-CR-1 PCa cells. The lung adenocarcinoma cell line H1563 was used as a positive control for mesothelin surface expression. Data are representative of two independent experiments. Percentages of positive cells are shown in the histograms. Grey trace: isotype control; blue trace: anti-mesothelin. (**D**) Western blot analysis of mesothelin expression in a panel of PCa cell lines.

Previous research has reported the rare expression of mesothelin in primary prostate tumors [53,54]. Immunohistochemical analysis of three primary PCa sections confirmed these findings, with weak immunostaining observed only in a small region of the primary tumor in a CRPC case, showing low staining intensity (Supplementary Figure S4). To clarify the expression of mesothelin in clinical samples, we thought to extent the analysis to metastatic samples. Given the scarcity of metastatic samples and the lack of routine clinical analysis of such samples, we investigated mesothelin (*MSLN*) mRNA levels using publicly available gene expression datasets of metastatic and primary PCa samples. Z-score comparisons revealed upregulation of *MSLN* levels in metastatic samples across several cohorts: 10% in the prad_MSKCC cohort (2/19), 6.7% in the prad_FHCRC cohort (10/149), and 4.2% in the prad_SU2C cohort (5/118) (Figure 1A). In contrast, *MSLN* levels were reduced or marginal in primary PCa cases in the prad_FHCRC cohort, with 0% (0/22), and with only 1.5% in the prad_MSKCC cohort (2/131) showing upregulated expression. In the WCMC cohort, comprising castration-resistant tumors, elevated MSLN expression was observed in 6.6% (1/15) of the CRPC-Neuroendocrine cases, and in 3% (1/34) of the CRPC-adeno cases, this later corresponding to one case of lymph node metastasis.

Collectively, these findings indicate that mesothelin is poorly expressed in primary PCa tumors but may be upregulated in metastatic PCa lesions.

### 3.2. A PCa Model with Aggressive Features Endogenously Expresses Significant Amounts of Mesothelin

We characterized *MSLN* expression in a panel of prostate cancer cell lines (Figure 1B). Androgen-sensitive LNCaP and VCaP cells, which are considered to have low metastatic potential, exhibited the lowest *MSLN* levels. In models of metastatic and CRPC tumors, intermediate levels were found in PC3 and 22Rv1 cells. *MSLN* levels were highest in DU145 and 22Rv1-CR-1 cells. Our previous work demonstrated that compared with their parental epithelial counterparts 22Rv1, 22Rv1-CR-1 cells have superior aggressiveness and strong mesenchymal attributes coinciding with enhanced capacities for migration, invasion, clonogenicity, and cell-autonomous growth [57]. This is mainly explained by overexpression of CRIPTO in these cells (also referred to as CR-1 or TDGF1), an embryonic factor associated with metastatic potential, cancer stem cell maintenance, and EMP [66,67].

Flow cytometry analysis indicated that 22Rv1-CR-1 cells expressed mesothelin on their cell membrane, unlike DU145 cells, wherein no evidence of cell surface expression was found (Figure 1C). Mesothelin reportedly exists in both mature and immature forms. Immunoblot analysis revealed a predominant band at approximately 40 kDa, corresponding to the mature form of mesothelin, in 22Rv1-CR-1 cells, with lower levels of the immature precursor (pre-mesothelin, ~69 kDa) detected (Figure 1D). Similarly, a subpopulation of 22Rv1-CR-1 cells, enriched for mesothelin through cell sorting (referred to as 22Rv1-CR-1-MESO), exhibited elevated levels of both the mature and precursor forms.

**Figure 2:**
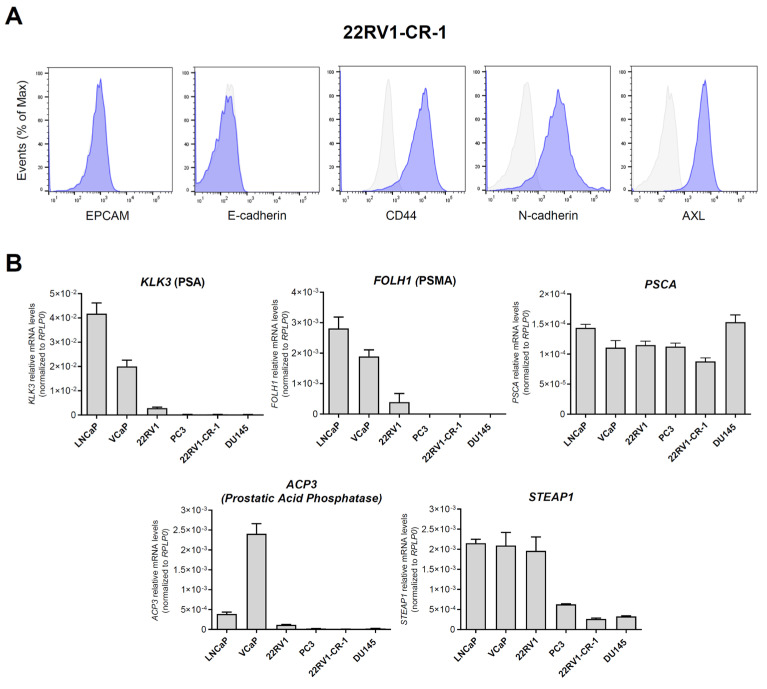
22Rv1-CR-1 cells exhibited mesenchymal characteristics and reduced expression of prostate differentiation markers. (**A**) The indicated markers were assessed by flow cytometry to determine the epithelial/mesenchymal status of 22Rv1-CR-1 cells. Grey trace: isotype control; blue trace: antibodies against indicated markers; (**B**) qRT-PCR analysis of prostate epithelial marker expression in 22Rv1-CR-1 and other PCa cell lines. mRNA levels were normalized to *RPLP0* expression. Data represent the mean ± SEM of triplicates from two independent experiments.

To further delineate the phenotype of 22Rv1-CR-1 cells, we then conducted flow cytometry and qRT-PCR analyses (Figure 2). The cells exhibited a mesenchymal-like phenotype marked by strong expression of typical mesenchymal markers (CD44, N-cadherin, and receptor tyrosine kinase AXL) along with the absence of epithelial markers (EPCAM and E-cadherin) (Figure 2A). qRT-PCR analysis also revealed low or absent expression of typical prostate differentiation markers in 22Rv1-CR-1 cells, such as *FOLH1* (PSMA), *KLK3*, *ACP3* (prostatic acid phosphatases), and *STEAP1*, in contrast to that found in other PCa cell lines (Figure 2B). These findings suggested that the 22Rv1-CR-1 model may be useful for evaluating therapeutic strategies targeting mesothelin for aggressive PCa cells with EMP features or reduced prostate differentiation.

### 3.3. Characterization of Meso-BBζ-CART Cells

**Figure 3:**
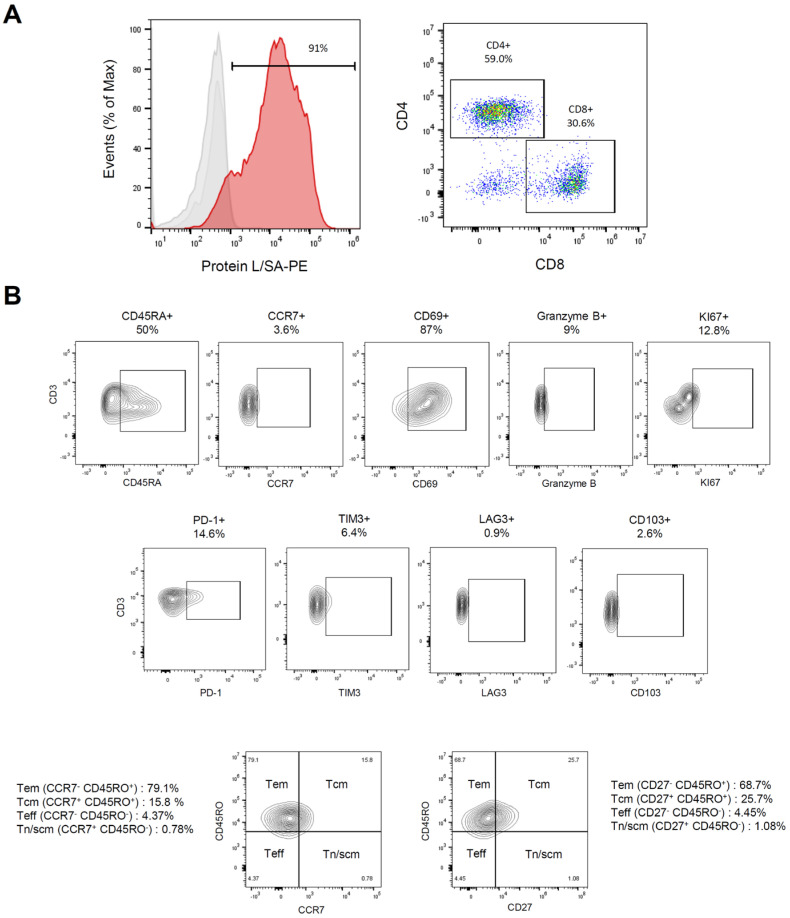
Characterization of Meso-BBζ-CART cells. (**A**) Flow cytometric analysis of CAR expression on transduced human T cells using protein L, which binds to the CAR scFv [70] (**left**); grey traces: unstained or SA-PE alone; red trace: Protein L followed by SA-PE; A representative cytometry plot depicting the percentage of CD4 versus CD8 T cells (**right**); (**B**) Phenotypic analysis of CAR-T cells after ex-vivo expansion, stained for markers of differentiation (CCR7, CD45RA, CD45RO, CD27), activation (CD69), cytotoxicity (Granzyme B), proliferation (Ki67), tissue residency (CD103), and exhaustion (PD-1, TIM3, LAG3). Data are representative of at least two independent experiments.

To evaluate whether aggressive PCa cells could be targeted by CAR-T cells, we utilized second-generation CAR-T cells targeting mesothelin (hereafter Meso-CART cells), which include the intracellular modules 4-1BB and CD3zeta. These CAR-Ts are known for their persistence in responding patients [55,68,69]. Flow cytometry analysis showed that 80–85% of the T cells expressed CARs after expansion, with a CD4/CD8 ratio of 1.9 (Figure 3A). A reduced proportion of cells expressed the inhibitory receptors PD-1 (14.6%), LAG-3 (0.9%), and TIM-3 (6.4%), suggesting the absence of an exhaustion phenotype (Figure 3B). In contrast, 89% of CAR-T cells expressed the activation marker CD69. The tissue-resident marker CD103 was not expressed. The T cell population had reduced CCR7 expression, indicating a paucity of naïve and central memory T cells. Approximately 50% of the cells expressed CD45RA, suggesting a predominance of effector T cells. Further analysis of memory differentiation was performed using CD45RO, in conjunction with CCR7 or CD27, to distinguish effector memory (Tem), central memory (Tcm), effector (Teff), naïve (Tn), and stem cell-like memory (Tscm) T cell populations. The results indicated a majority of Tem-like cells (68–79%), along with a notable fraction of Tcm-like cells (15–26%).

### 3.4. Meso-BBζ-CAR-T Cells Efficiently Kill Aggressive PCa Cells Expressing Mesothelin

**Figure 4:**
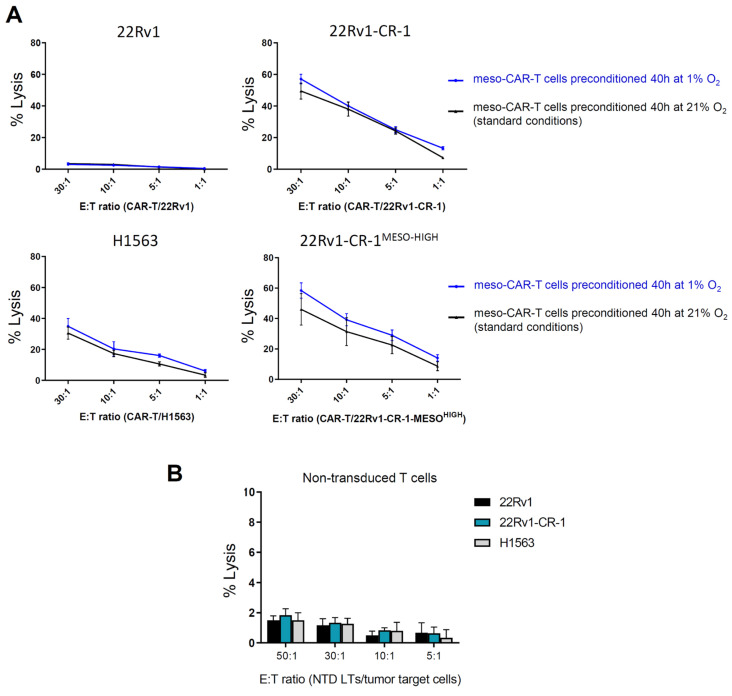
Meso-BBζ-CART cells efficiently lyzed mesothelin-expressing PCa cells. (**A**) Meso-CART cells preincubated under hypoxia (blue line) or normoxia (black line) conditions for 40 h, then co-cultured for 4 h with target carcinoma cells at various E:T ratios. Experimental workflow is presented in Supplementary Figure S1. Specific lysis of cancer cells was determined by a standard radioactive cytotoxic assay. The 22Rv1 cells (mesothelin-negative) served as negative controls, while the H1563 cells (mesothelin-positive) served as positive controls. The 22Rv1-CR-1-MESO cells represent a mesothelin-enriched subpopulation of 22Rv1-CR-1. Data are shown as mean ± SEM of triplicates and representative of two independent experiments. (**B**) Cytotoxicity of non-transduced T cells under normoxic conditions, shown as mean ± SEM.

We assessed meso-CAR-T cell cytotoxicity against different target cell lines using a chromium-51 release lysis assay (Supplementary Figure S1). The effector cells (E), Meso-CAR-T cells (or non-transduced control T cells) were first cultured for 40 h under standard normoxic (21% O_2_) or hypoxic (1% O_2_) conditions. They were then co-cultured for 4 h with the target cells (T = tumor line), expressing or not expressing the tumor-associated antigen mesothelin. Cell-mediated lysis % was calculated from the release of chromium from dead cancer cells into the medium. At the different effector-to-target (E/T) ratios tested, CAR-T cells showed strong killing capacities against mesothelin-expressing cells (22Rv1-CR-1, 22Rv1-CR-1-MESO, and H1563) (Figure 4A), reaching 40–50% lysis in only a few hours at the highest E:T ratio of 30:1. Comparable results were obtained when CAR-T cells were preconditioned under normoxic or hypoxic conditions. Even at 1:1 E/T ratio, the meso-CAR-T cells efficiently killed more than 5–10% of the 22Rv1-CR-1 PCa cells. In contrast, meso-CAR-T cells showed minimal activity against mesothelin-negative 22Rv1 cells, with <2% lysis measured at all E:T ratios, that is, below the sensitivity threshold for this assay.

Likewise, non-transduced T cells, serving as negative control, exhibited negligible activity (i.e., <2% lysis) against the different target cancer cells, regardless of their mesothelin expression status (Figure 4B).

Collectively, these findings suggest that meso-CAR-T cells can eliminate aggressive PCa cells in an antigen-specific manner. Notably, mesothelin-CAR-T cells demonstrated comparable cytotoxicity toward both 22Rv1-CR-1 and 22Rv1-CR-1-MESO cells, indicating that variations in tumor antigen density, which is higher in 22Rv1-CR-1-MESO, did not significantly influence killing efficiency in this assay investigating second generation CAR-T cells. These results were consistent under both normoxic and hypoxic preconditioning.

### 3.5. Meso-BBζ-CAR-T Cells Showed Similar Cancer Killing Capacities in Normoxia and Hypoxia

**Figure 5:**
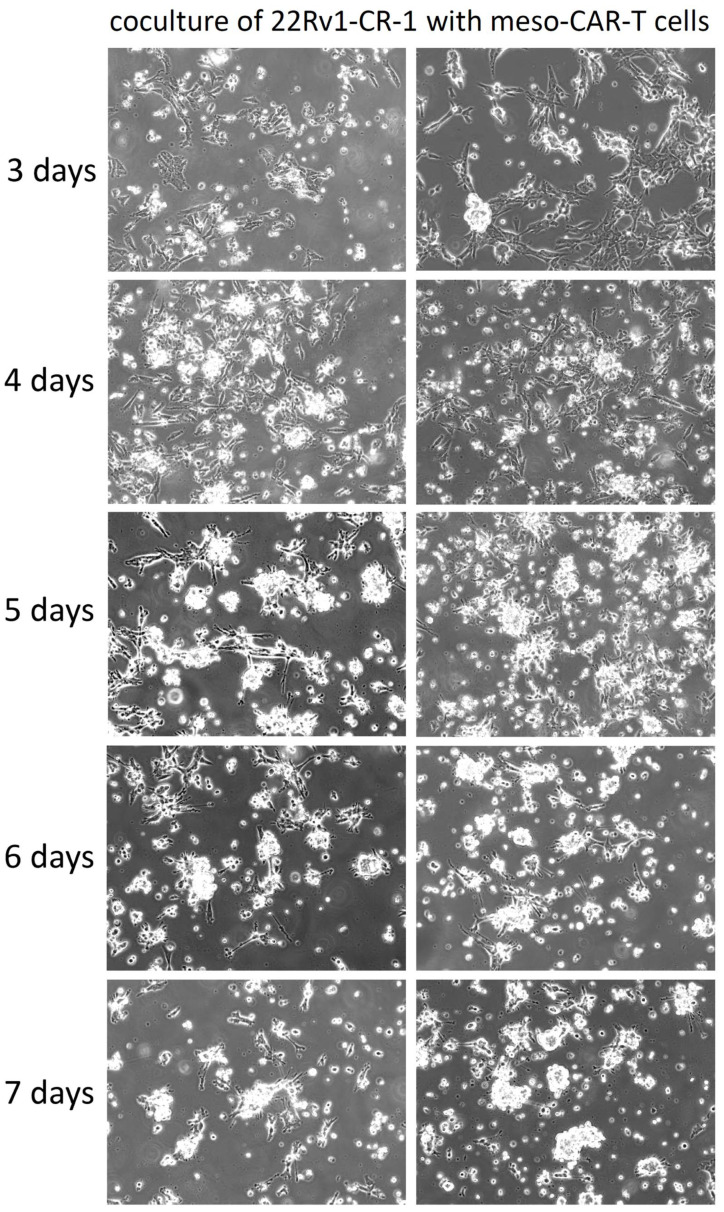
Meso-BBζ-CART cells demonstrated killing capacity in long-term co-culture assays. Phase-contrast microscopy images of 22Rv1-CR-1 cells co-cultured with meso-CAR-T cells for up to 7 days at an initial E:T ratio of 1:5. Magnification: 200×.

We performed tumor-killing assays by co-culturing effector cells with a monolayer of target carcinoma cells for up to 7 days at an initial effector-to-target (E:T) ratio of 1:5, favoring tumor cells. Under these conditions, meso-CAR-T cells exhibited robust cytotoxicity against mesothelin-expressing 22Rv1-CR-1 cells, leading to near-complete tumor cell eradication by day 7 (Figure 5).

**Figure 6:**
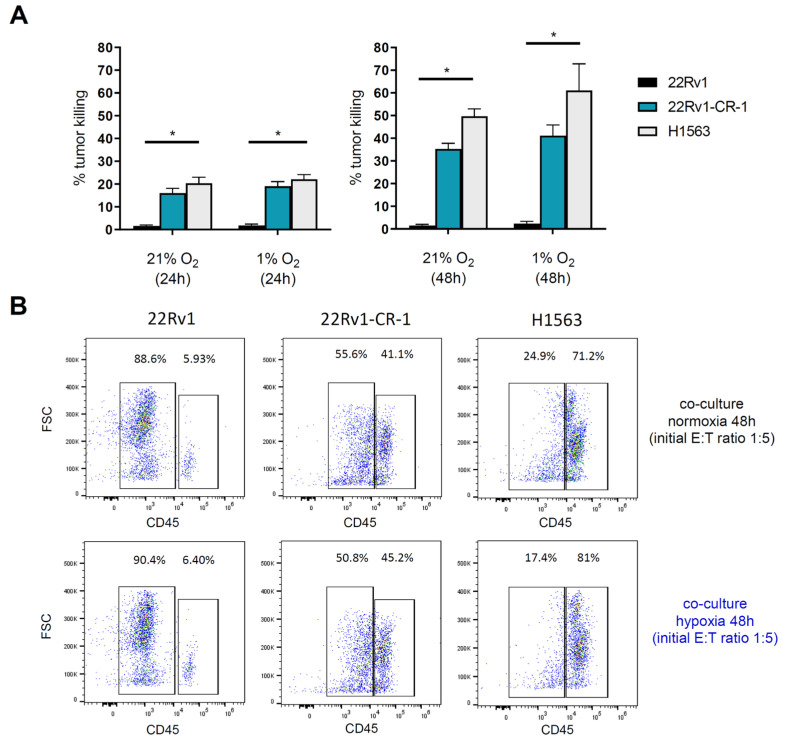
Meso-BBζ-CAR-T cells exhibit similar killing capacity under normoxic and hypoxic conditions. (**A**) Bar graphs showing tumor cell killing after 24 and 48 h of co-culture at various E:T ratios. Experimental workflow is presented in Supplementary Figure S3. Two-way ANOVA revealed a significant cell line effect (*p* < 0.0001), but no significant oxygen level or interaction effects. Multiple comparisons indicated significant differences as follows: normoxia: 22Rv1 vs. 22Rv1-CR-1 (*p* = 0.0002 at 24 h; *p* = 0.017 at 48 h); 22Rv1 vs. H1563 (*p* < 0.0001 at both time points); hypoxia: 22Rv1 vs. 22Rv1-CR-1 (*p* < 0.0001 at 24 h; *p* = 0.0006 at 48 h); 22Rv1 vs. H1563 (*p* < 0.0001 at both time points). The significant effects are recapitulated in the figure * (*p* ≤ 0.05). (**B**) Representative flow cytometry dot plots comparing Meso-BBζ-CAR-T cell activity under normoxic and hypoxic conditions at 24 and 48 h.

**Figure 7:**
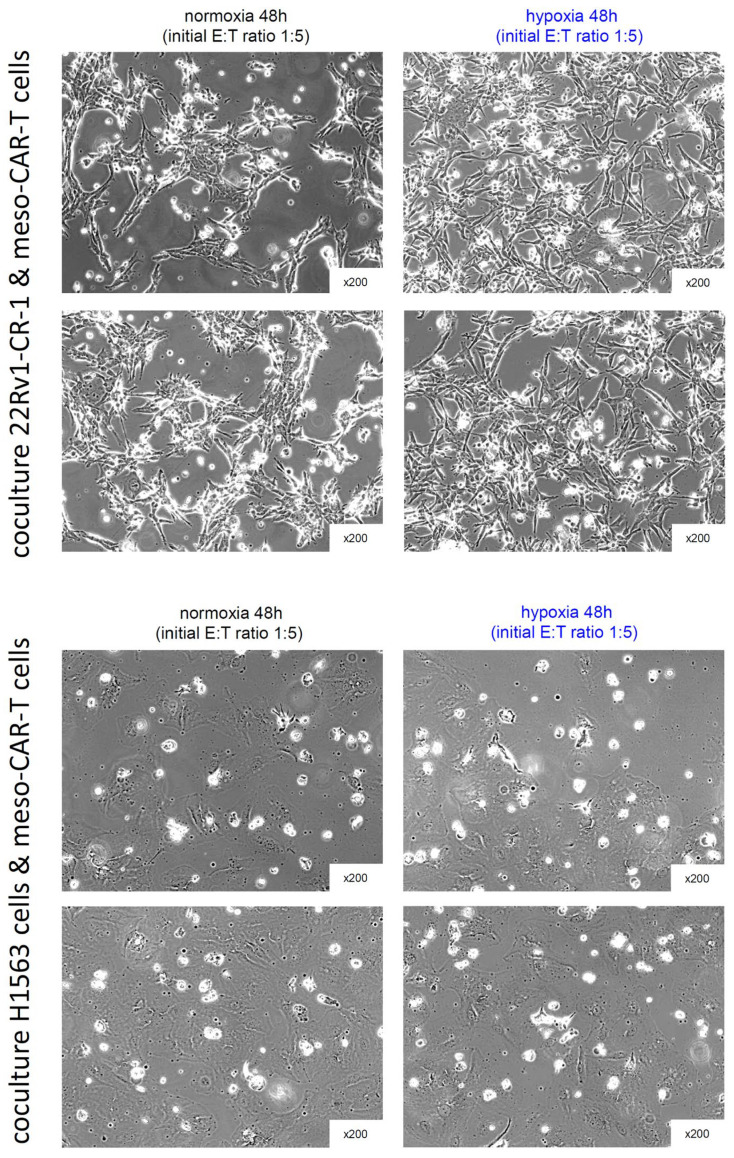
Phase-contrast microscopy of 22Rv1-CR-1 and H1563 cells co-cultured with meso-BBζ-CAR-T cells. Images were taken after 48 h of co-culture, as described in Figure 6 and Supplementary Figure S3. Magnification: 200×.

We further assessed the cytotoxic activity of meso-CAR-T cells under normoxic and hypoxic conditions, quantifying the mortality rates at 24 h and 48 h (Supplementary Figure S3). Under these conditions, meso-CAR-T cells demonstrated significant tumor cell killing (Figure 6A), with mortality rates of 15% at 24 h and 40% at 48 h for 22Rv1-CR-1 cells. For H1563 lung carcinoma cells, mortality reached 20% at 24 h and 50% at 48 h (Figure 6A,B and Figure 7). Comparable levels of cytotoxicity were observed under normoxic and hypoxic conditions, although a slight trend toward increased killing under hypoxia was noted at the 48 h time point. Importantly, no cytotoxic activity was detected against mesothelin-negative parental 22Rv1 cells, confirming the antigen specificity of mesothelin-CAR-T cells under hypoxia (Figure 6A,B).

**Figure 8:**
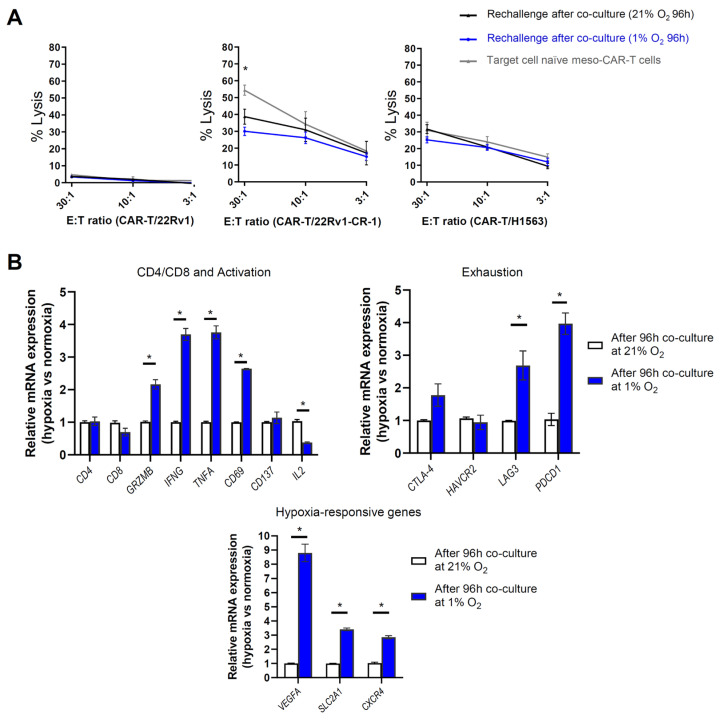
Prolonged hypoxia induces activation and exhaustion markers in mesothelin-CAR-T cells. (**A**) Meso-CAR-T cells were cultured with target carcinoma cells for 96 h under hypoxia (blue line) or normoxia (black line), then re-challenged with fresh target cells under normoxia for 4 h at various E:T ratios. Cytolytic activity was assessed using a standard radioactive assay (see experimental workflow shown in Supplementary Figure S2). Naïve (antigen-unexposed) CAR-T cells were used as controls. The 22Rv1 cells served as mesothelin-negative controls; the H1563 cells were positive controls. Data represent mean ± SEM of triplicates from two independent experiments. Two-way ANOVA results indicated CAR-T group effect: Not significant; E:T ratio effect: 22Rv1 (*p* = 0.02); 22Rv1-CR-1 and H1563 (*p* < 0.0001); Interaction effect: 22Rv1 and H1563 (not significant); 22Rv1-CR-1 (*p* = 0.047); Pairwise comparisons: rechallenged normoxic CAR-T vs. naïve CAR-T (22Rv1-CR-1, *p* = 0.021 at E:T 30:1 (* *p* ≤ 0.05 on graph); not significant at other ratios); rechallenged hypoxic CAR-T vs. naïve CAR-T (22Rv1-CR-1, *p* = 0.001 at E:T 30:1; not significant at other ratios). (**B**) qRT-PCR analysis of gene expression in meso-CAR-T cells co-cultured with target carcinoma cells under normoxic or hypoxic conditions for 96 h. Data represent the mean ± SEM of triplicates from two independent experiments. Statistical significance was evaluated using the Mann–Whitney test (* *p* ≤ 0.05).

To further explore the impact of hypoxia on the cytotoxic potential of meso-CAR-T cells, we conducted additional experiments in which meso-CAR-T cells were first co-cultured with mesothelin-expressing target cells for 96 h in either normoxic or hypoxic conditions. Subsequently, the CAR-T cells were rechallenged with fresh target cells and subjected to cytotoxicity assays to assess their lytic capacity (Supplementary Figure S2). As a control, antigen-naïve meso-CAR-T cells cultured under standard conditions were also included in these experiments to evaluate potential exhaustion effects. Under these conditions, meso-CAR-T cells demonstrated cytotoxicity against antigen-expressing 22Rv1-CR-1 cells, with killing ranging from 15% to 40%, depending on the E:T ratio (Figure 8A). Similar results were observed when the cells were exposed to antigen-expressing target cells in either hypoxic or normoxic conditions. As expected, meso-CAR-T cells failed to lyze 22Rv1 parental cells, which lack mesothelin expression. Naïve meso-CAR-T cells exhibited a trend toward greater cytolytic activity compared with antigen-exposed meso-CAR-T cells, but this effect was only observed at the highest E:T ratio of 30:1, not at the 10:1 or 3:1 ratios. Moreover, this observation was restricted to 22Rv1-CR-1 cells, as meso-CAR-T cells effectively killed mesothelin-positive H1563 lung cancer cells in a similar manner under both normoxic and hypoxic conditions. In summary, these data suggest that meso-CAR-T cells could selectively and potently kill mesothelin-expressing carcinoma cells, with no significant impact of hypoxia on their cytotoxic activity.

To further investigate the potential influence of hypoxia on meso-CAR-T cell functionality, we analyzed changes in activation and exhaustion markers following co-culture with target cells under both normoxic and hypoxic conditions. Meso-CAR-T cells were co-cultured with mesothelin-expressing target cells for 96 h, and expression levels of various markers were assessed by RT-qPCR. The analysis revealed upregulation of markers associated with T cell activation and cytotoxic activity, including *Granzyme B*, *IFNG*, *TNFA*, and *CD69*, along with a reduction in *IL2* expression (Figure 8B). The expression of CD4, CD8, and CD137 remained unchanged. Moreover, hypoxia induced the expression of exhaustion marker genes *PDCD1* (encoding for PD-1) and *LAG3* by 2 to 4 thresholds, with a modest increase in *CTLA4* expression, while *HAVCR2* (encoding for TIM3) remained unaffected. Additionally, analysis of hypoxia-inducible genes, such as *VEGFA*, *CXCR4*, and *SLC2A1* (encoding for GLUT1), confirmed the hypoxic status of the cells under hypoxic conditions compared with normoxia.

These findings suggest that hypoxic stress may enhance CAR-T cell activation while concomitantly upregulating certain inhibitory receptors. This dual effect may contribute to comparable cytolytic activity against target tumor cells in both normoxic and hypoxic environments.

## 4. Discussion

Metastatic PCa remains a largely incurable condition, with most patients facing limited therapeutic options once resistance to anti-androgen therapies has developed. The high degree of heterogeneity and plasticity in aggressive prostate cancers, coupled with a complex tumor microenvironment, underscores the critical need for novel therapeutic strategies. Cell-based therapies such as CAR-T therapy offer considerable promise; however, further improvements are necessary to optimize their efficacy. Expanding the repertoire of targetable antigens and advancing our understanding of CAR-T cell dynamics under stress conditions imposed by the tumor microenvironment are key steps forward.

In this study, we characterized mesothelin expression in prostate cancer and identified a relevant cancer cell line model for investigating mesothelin-targeted CAR-T therapy in this context. Using this model, we demonstrated that mesothelin-targeted CAR-T cells can exert potent cytotoxic effects against PCa cells with aggressive features, suggesting the potential of mesothelin as a therapeutic target in this disease.

To enhance clinical relevance, our study focused on second-generation CARs incorporating a 4-1BB costimulatory domain. Alternative constructs, including other second-generation and third-generation CARs, alone or in combination with other approaches, may also hold therapeutic potential and warrant further investigation.

Due to the limited availability of metastatic PCa tissue samples, we analyzed mesothelin mRNA expression in publicly available datasets, prioritizing those with both primary and metastatic samples. In line with previous studies, we found that mesothelin expression is infrequent in primary prostate tumors [47,48], but shows increased expression in metastatic samples. This suggests that mesothelin-targeted therapies may be most appropriate for metastatic PCa. Additional studies with expanded cohorts are needed to validate these findings, assess mesothelin mRNA and protein expression in greater detail, and further elucidate if it has a role in the metastatic process. Only a subset of patients would be expected to express mesothelin at sufficient levels for effective targeting. We hypothesize that metastatic PCa patients with poorly differentiated tumors, marked by reduced AR activity and low neuroendocrine differentiation, a mesenchymal-like phenotype, may represent this mesothelin-expressing subgroup [71]. Supporting this hypothesis, mesothelin expression was notably higher in AR-deficient cell lines DU145 and PC3 than in AR-active cells (LNCaP, VCaP, and 22Rv1), suggesting an inverse correlation between mesothelin expression and the AR signaling. Further work is needed to identify patients in this subgroup, and to develop robust methods for assessing mesothelin expression in metastatic PCa patients. It is also worth noting that DU145 expresses some levels of mesothelin mRNA, but failed to express detectable levels of the protein at the cell surface, unlike 22Rv1-CR-1, suggesting differential protein trafficking or post-transcriptional regulation between these two carcinoma cell lines.

In 2D co-culture assays (4 h to 5 days), mesothelin-CAR-T cells with a second-generation construct exhibited high specificity for 22Rv1-CR-1 cells, an aggressive PCa model. The 22Rv1-CR-1 cell line, an aggressive variant of castration-resistant 22Rv1 cells, exhibits pronounced mesenchymal features, and substantial membrane-bound mesothelin expression, which remains stable across cell passages and under hypoxic conditions. Enriching mesothelin-expressing cells via cell sorting did not significantly change tumor cell killing in our assays, corroborating previous studies that highlight the high sensitivity of CAR-T cells compared with alternative strategies, such as antibody–drug conjugates or bi-specific and naked antibodies, which may require higher antigen levels to achieve comparable efficacy [72,73].

Our assays revealed minimal differences in cytolytic capacity between normoxic and hypoxic conditions, despite slight variations in susceptibility of the 22Rv1-CR-1 and H1563 lines. Nevertheless, signs of CAR-T cell exhaustion (elevated *LAG3* and *PDCD1*, reduced *IL2*), along with increased expression of cytotoxic and activation markers (*GZMB*, *CD69*, *IFNG*, *TNFA*), suggest the need for further investigation. Long-term co-culture in hypoxia may introduce bias due to factors such as differences in media composition, differential nutrient consumption by carcinoma cells, or absence of exogenous IL-2. Future in vivo studies that more closely mimic human prostate cancer pathology and incorporate these parameters will be critical to advancing CAR-T therapy. While we utilized mesothelin-BBζ-CAR-T cells, it remains to be determined if T cells engineered with different CAR constructs might respond differently to hypoxic conditions.

Among the prostate cancer models investigated in this study, 22Rv1-CR-1 was the only one expressing mesothelin at significant levels at the cell surface, which we recognize as a limitation of our study. This 22Rv1-CR-1 model was originally established to represent features of aggressive prostate cancer. Although CRIPTO expression in this line exceeds that of most clinical samples, it remains within the range seen in a subset of aggressive PCa [57,74,75]. Notably, in two of four gene expression datasets (PRAD-MSKCC and PRAD-SU2C), a strong correlation was observed between *MSLN* and *TDGF1* (alias CRIPTO) z-scores (*p* < 0.0001) (Supplementary Figure S5), warranting further investigation. Similarly, the potential causal relationship between an EMP-like phenotype and mesothelin expression in this cancer model remains unexplored, despite evidence in lung cancer and mesothelioma indicating that mesothelin promotes epithelial-to-mesenchymal transition and tumorigenicity in human lung cancer and mesothelioma models [76].

Given the antigenic heterogeneity of prostate tumors, it is likely that targeting multiple antigens will be necessary to achieve complete eradication of aggressive tumors. Thus, ongoing identification of new targets remains crucial. In our analysis, prostate-associated markers including PSMA, PSA, prostate acid phosphatase, and STEAP1 were preferentially expressed in cell lines with an active AR pathway (LNCaP, VCaP, and 22Rv1). STEAP1 levels remained relatively high even in AR-deficient cells (DU145, 22Rv1-CR-1, and PC3), while PSCA was expressed similarly across all tested cell lines. These findings suggest that these cell surface markers could serve as valuable targets in CAR-T therapeutic strategies, with consideration of the molecular and pathological subtypes of prostate cancer. In addition, combining CAR-T therapy with other treatment or immunomodulatory approaches may further enhance the therapeutic efficacy and persistence of CAR-T therapies [77,78,79].

In addition to efficacy, enhancing toxicity management should be an area of intense research. As discussed, PCa cases and PCa cell lines demonstrated low to intermediate levels of mesothelin expression. This level of expression appears well-situated for CAR-T therapy, which, unlike other strategies, does not require overexpression of the target antigen for therapeutic efficacy. Thus, improving monitoring of mesothelin expression may be needed to guide the choice of treatment, identify optimal candidates for CAR-T treatment, and proactively address potential toxicities.

In normal tissue, mesothelin is expressed primarily in non-vital organs such as the thymus, seminal vesicles, gallbladder, uterus, and placenta [54]. Expression has also been noted in the gastrointestinal tract, as supported by Weindemann et al., but with low-density of positive cells, suggesting a reduced risk of severe off-target effects. Clinical trials testing second-generation CAR-T cells targeting mesothelin have demonstrated a favorable safety profile, with only a few instances of severe toxicity reported [52,80]. Specific patient groups, including those with pulmonary abnormalities or underlying inflammatory or fibrotic conditions may warrant additional caution [81]. Encouragingly, CAR-T toxicities are increasingly well managed by clinical teams as their experience and post-treatment care protocols evolve.

## 5. Conclusions

Advancing chimeric antigen receptor (CAR) T cell therapy for solid tumors, such as aggressive prostate cancer (PCa), is challenging due to tumor heterogeneity and complex microenvironments. Mesothelin has emerged as a promising target for CAR-T therapy in solid tumors. In this study, we analyzed gene expression datasets and evaluated mesothelin expression across PCa gene expression datasets and preclinical models. We found significant mesothelin enrichment in 4–10% of metastatic PCa tumors and in a PCa model with marked epithelial–mesenchymal plasticity features. In experimental studies, second-generation meso-CAR-T cells exhibited strong cytotoxicity and selectivity towards the mesothelin-expressing PCa cells under normoxic and hypoxic conditions, suggesting their potential as a targeted therapy for metastatic PCa.

## Data Availability

The original contributions presented in this study are included in the article/Supplementary Material. Further inquiries can be directed to the corresponding author.

## Supplementary Materials

The following supporting information can be downloaded at https://www.mdpi.com/article/10.3390/biomedicines13051215/s1: Supplementary Figure S1: Experimental workflow for cytotoxicity assays shown in Figure 4; Supplementary Figure S2: Experimental workflow for rechallenge assays shown in Figure 8; Supplementary Figure S3: Experimental workflow for tumor cell killing assays shown in Figure 6 and Figure 7; Supplementary Figure S4: Mesothelin expression by IHC and IF; Supplementary Figure S5: Correlation analysis between *MSLN* and *TDGF1* gene expression in various PCa datasets.
