# Supplementary material for: Targeting Aggressive Prostate Carcinoma Cells with Mesothelin-CAR-T Cells
Source: Biomedicines. 2025 May 16;13(5):1215. doi: 10.3390/biomedicines13051215 (PMC12109071; doi:10.3390/biomedicines13051215)
Supplement: Supplementary file 1 [file biomedicines-13-01215-s001.zip › biomedicines-3603137-supplementary.pdf]

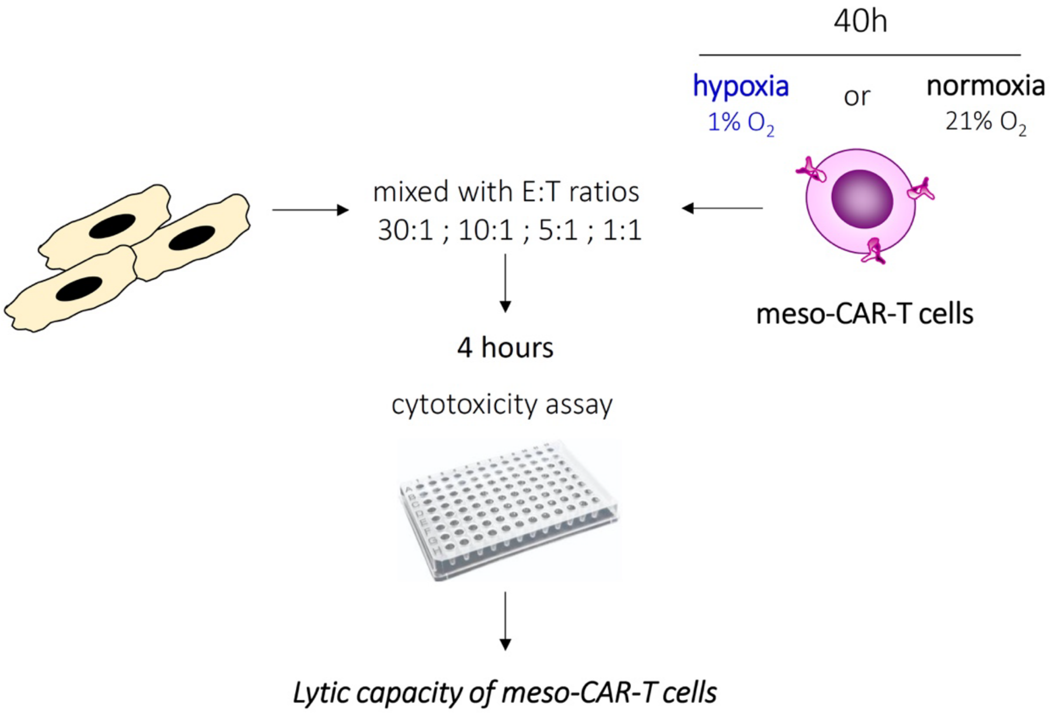

Figure S1: *Experimental workflow for cytotoxicity assays shown in* ***Figure 4***

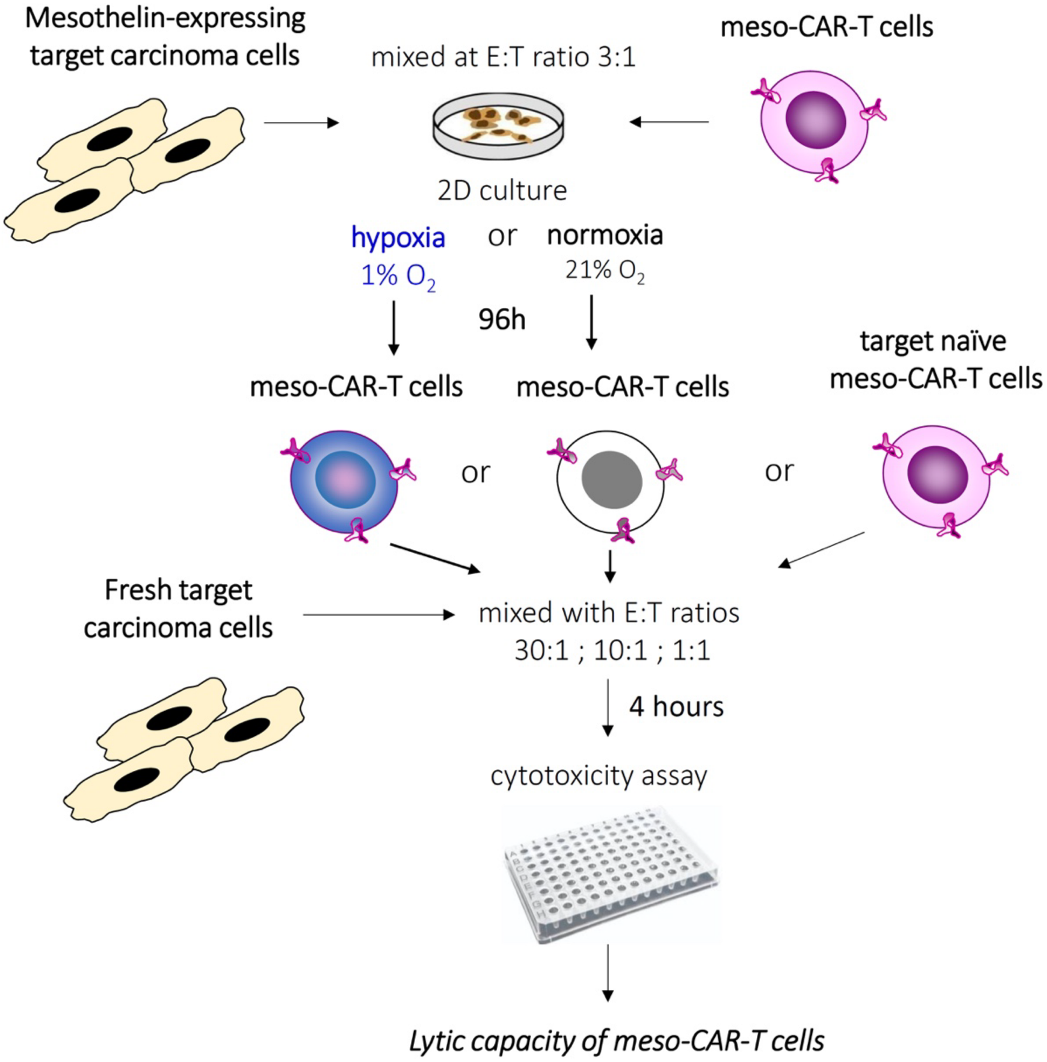

Figure S2: *Experimental workflow for rechallenge assays shown in* ***Figure 8***

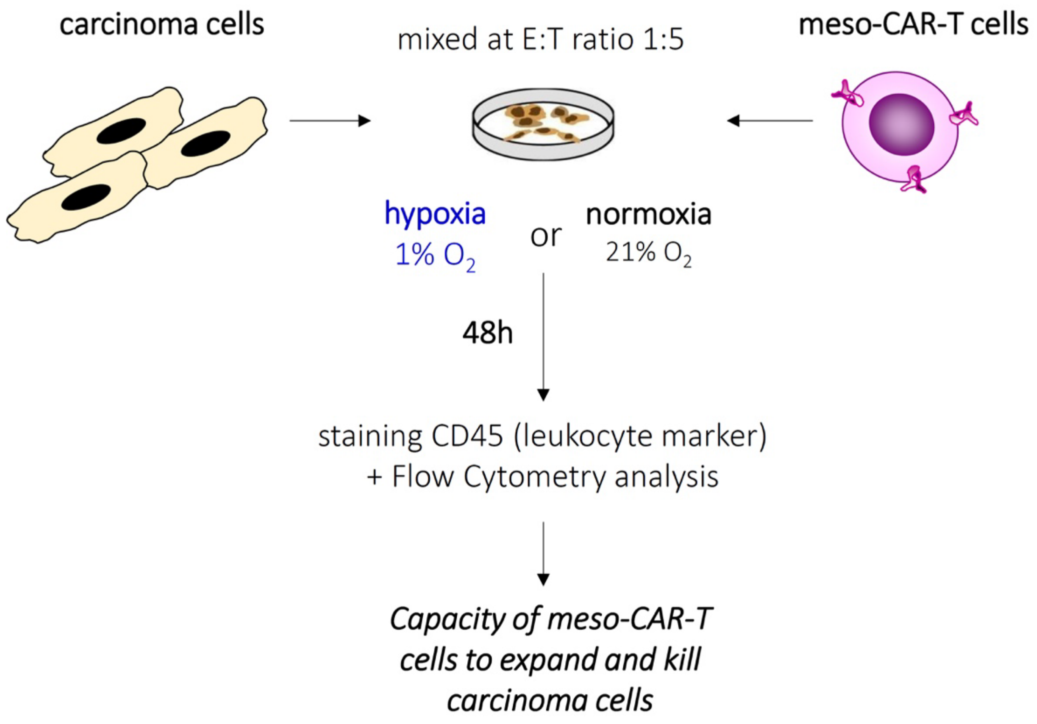

Figure S3*: Experimental workflow for tumor cell killing assays shown in* ***Figures 6*** *and* ***7***

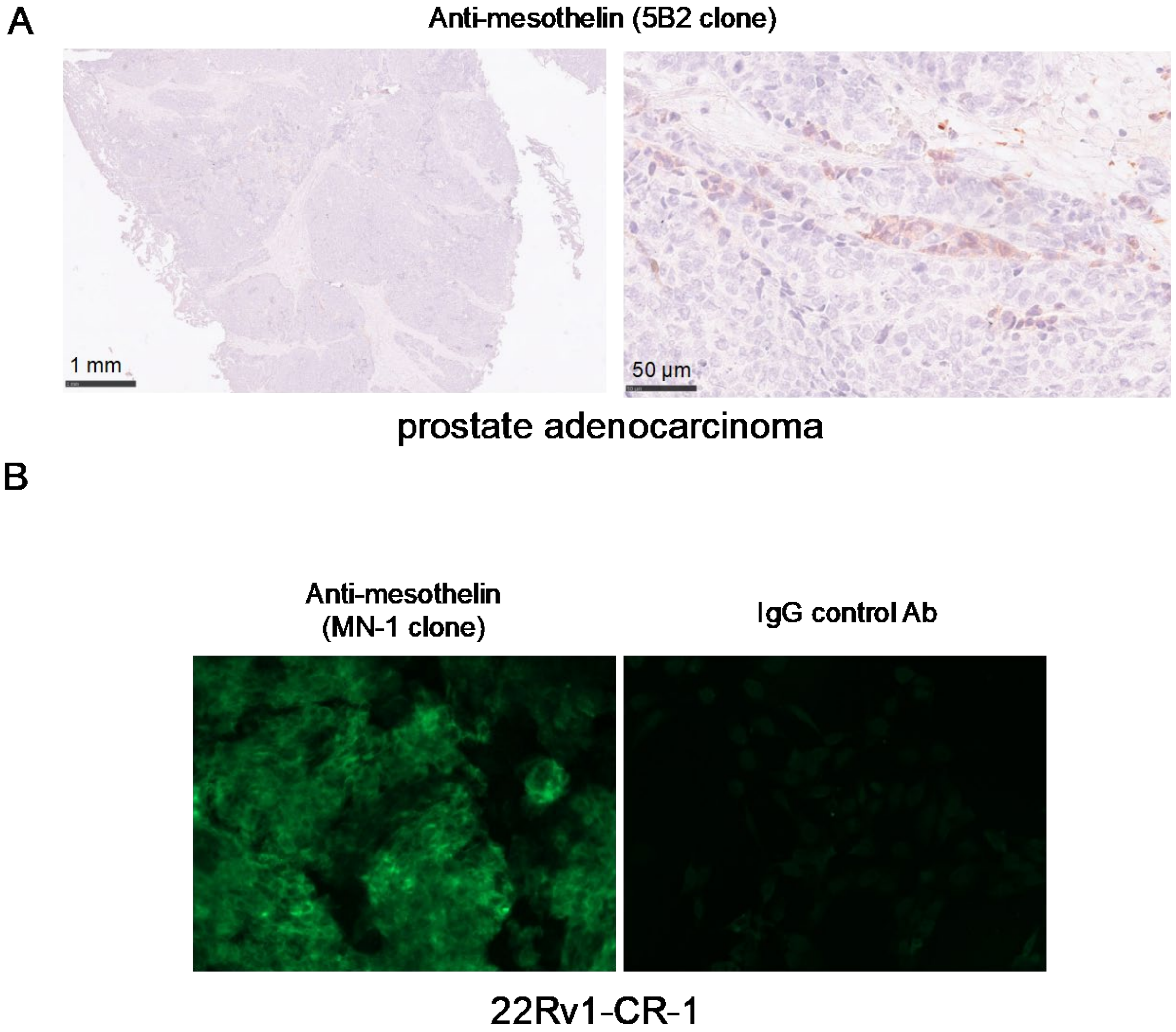

Figure S4: *Mesothelin expression by IHC and IF. A) Representative prostate cancer case with heterogeneous and low MSLN expression (right panel), with absent expression in most tumor foci (left panel). B) Mesothelin expression in 22Rv1-CR-1 cells assessed by immunofluorescence.*

MSLN* vs. *TDGF1	**prad_MSKCC (all cases)**	**prad_MSKCC (metastatic cases)**	**prad_SU2C**	**prad_FHCRC (all cases)**	**prad_FHCRC (metastatic cases)**	**CRPC_WCMC**
**Pearson r**	**0.4708**	0.365	**0.6064**	-0.04799	-0.06007	0.05862
95% confidence interval	**0.3358 to 0.5868**	-0.1069 to 0.7027	**0.4780 to 0.7094**	[illegible].1966 to 0.102	-0.2188 to 0.1017	-0.2263 to 0.3343
R squared	**0.2216**	0.1333	**0.3677**	0.002303	0.003608	0.003436
P value	**<0.0001**	0.1244	**<0.0001**	0.5331	0.4668	0.6891
P (two-tailed)	****	ns	****	ns	ns	ns
P value summary	**Yes**	No	**Yes**	No	No	No
Number of XY Pairs	**150**	19	**118**	171	149	49

Figure S5: *Correlation analysis between MSLN and TDGF1 gene expression in various PCa datasets.*
